# Endodontic Microbiome of Fractured Non-vital Teeth in Dogs Determined by 16S rRNA Gene Sequencing

**DOI:** 10.3389/fvets.2019.00348

**Published:** 2019-10-09

**Authors:** Marjory Xavier Rodrigues, Ana Nemec, Nadine Fiani, Rodrigo C. Bicalho, Santiago Peralta

**Affiliations:** ^1^Department of Population Medicine and Diagnostic Sciences, Cornell University, Ithaca, NY, United States; ^2^Small Animal Clinic, Veterinary Faculty, University of Ljubljana, Ljubljana, Slovenia; ^3^Department of Clinical Sciences, Cornell University, Ithaca, NY, United States

**Keywords:** fractured teeth, non-vital teeth, primary endodontic infection, microbiome, dogs

## Abstract

Dental fractures resulting in pulp exposure will lead to an endodontic infection with microbes from the oral cavity. However, data on the endodontic microbial composition in veterinary dentistry is lacking. The aim of this study was to examine the microbiome of naturally occurring primary endodontic infections in client-owned dogs. The endodontic microbiome of 10 non-vital teeth with exposed pulp cavities was assessed using a 16S rRNA gene sequencing approach. The results were compared to the microbiome of the subgingival plaque of the same teeth. Analysis revealed an abundant mixed microflora of a comparable richness and diversity and with mostly the same phyla obtained from sulcal and endodontic samples. However, further analysis revealed significant differences between sulcal and endodontic samples in the relative abundance of the most abundant phyla and genera, with the relative abundance of Bacteriodetes being significantly higher in endodontic samples. Although each sample presented a particular profile regarding the genera identified, *Bacteroides* was the most abundant genus in the endodontic samples. *Snowella* was also significantly more abundant in endodontic samples, while *Porphyromonas* and *Fusobacterium* were significantly more abundant in sulcal samples. We confirmed that the microbiome of the diseased endodontic system is comparably abundant with microorganisms to the healthy subgingival plaque indicating that previous culture-based studies of primary endodontic infections in dogs underestimated the richness and diversity of the endodontic microbiota.

## Introduction

Dental fractures in dogs are very common, often resulting in exposure of the dental pulp to the oral cavity (1). The oral cavity and the gingival sulcus harbor highly diverse microbiota that can act as a source of microorganisms that can infect the compromised endodontic system (2–5). Primary endodontic infections are polymicrobial in nature and well-documented in humans (4, 6, 7), but much less so in dogs (8–10). Moreover, data obtained to date in veterinary dentistry stem from culture-based microbiological studies, which are known to underestimate the diversity of microbiota associated with endodontic disease in humans (4, 6, 7).

Due to the high incidence of tooth fractures (1), naturally occurring primary endodontic infections are of great medical and welfare concern in veterinary medicine. Thus, there is need for the development of better diagnostic and treatment strategies. A thorough understanding of the endodontic microbial composition and the resulting host response is therefore essential (11).

The aims of this study were to examine the microbiome of naturally occurring primary endodontic infections in dogs using a 16S rRNA gene sequencing approach, and to compare the results to the already well-characterized microbiome of the healthy subgingival plaque (2, 5). We hypothesized that the endodontic microbiome would be as rich and diverse as the subgingival microbiome with certain microorganisms (putative pathogens) enriched in endodontically diseased teeth.

## Materials and Methods

### Study Animals and Sample Collection

Study animals consisted of 10 systemically healthy adult dogs that were presented to Cornell University's Small Animal Dentistry and Oral Surgery Service for endodontic disease. Each patient was diagnosed with pulp exposure (complicated crown fracture due to trauma or wear) and a necrotic pulp of at least one canine tooth. The affected teeth were otherwise clinically and radiographically periodontally healthy. Animals that had received systemic immunosuppressive and/or systemic or oral topical antibiotic therapy in the previous 4 weeks were excluded. The diagnosis and experimental sample collection were performed by a board-certified veterinary dentist (SP) during general anesthesia prior to receiving standard-of-care intervention (i.e., periodontal treatment and extraction or endodontic treatment); anesthesia was supervised by a board-certified veterinary anesthesiologist. Only one affected canine tooth was sampled per animal; if more than one canine tooth was affected, the sampled tooth was chosen randomly. Relevant radiologic signs associated with the sampled tooth were noted including the presence of apical periodontitis (i.e., periapical lucency and/or inflammatory root resorption). All animals were inspected for the presence of intra- or extraoral draining tracts, soft tissue swelling, or cellulitis associated with the sampled tooth. Two independent samples were obtained aseptically from affected teeth. One sample (endodontic sample) was collected from the pulp cavity by inserting a 60 mm sterile endodontic paper point ranging between ISO #15 and #25, via the pulp exposure site all the way to the apical third while avoiding contact with any external dental or oral mucosal surface. Following previously reported protocols in similar veterinary studies, no dental dam was used (8, 10). Once inside the canal, the paper point was left in place for approximately 15 s prior to removal. The other sample (sulcal sample) was obtained by inserting a sterile endodontic paper point into the gingival sulcus of the same tooth and gently rubbing it along at least half of its circumference. All samples were obtained prior to any instrumentation or disinfection, or systemic antibiotic administration. Samples were collected, labeled, and stored individually in 1.5 ml sterile polypropylene microcentrifuge tubes, placed on ice, transported to the laboratory within 4 h, and then frozen at −80°C until analyzed.

### DNA Extraction, DNA Amplification, Library Preparation, and 16S rRNA Gene Sequencing

For DNA extraction, 1.0 mL of UltraPureTM distilled water (DNAse and RNAse free, Invitrogen, Grand Island, NY) was added to each microcentrifuge tube containing the paper point. The tubes were vortex-mixed for 10 min at room temperature. Subsequently, paper points were removed, and the tubes were centrifuged for 5 min at 13,000 rpm at room temperature, and the pellet obtained was used for DNA extraction. The DNeasy® PowerFood® Microbial Kit (Qiagen, Hilden, Germany) was used for DNA extraction following the manufacturer's instructions. Positive (*Staphylococcus aureus*, ATCC 25923) and negative (blank) controls were included in all steps performed. Primers 515F and 806R were used for amplification of the V4 hypervariable region of the bacterial/archaeal 16S rRNA gene and optimized for the Illumina MiSeq platform as previously described (12, 13). Amplicons obtained were standardized to the same concentration and pooled in order to obtain an equimolar library, which were sequenced using MiSeq reagent kit V2 for 300 cycles on the MiSeq platform (Illumina, Inc., San Diego, CA, USA).

### DNA Library Analysis and Statistical Analysis

The 16S rRNA gene sequences from the MiSeq platform were processed through the open-source software pipeline Quantitative Insights into Microbial Ecology (QIIME) (14). Sequences were filtered based on quality as previously described (15, 16) and binned into operational taxonomic units (OTUs) with 97% identity using UCLUST (17) against the Greengenes reference database. Using OTUs information, the richness (Chao1 index) and diversity (Shannon index) indices were calculated as described in a previous study (16) and compared between groups of samples evaluated.

The descriptive analysis was performed using JMP Pro 11 (SAS Institute Inc., Cary, NC) and the graphs presenting the relative abundance (%) of the most abundant genera and phyla of each sample were built using GraphPad Prism 8.2.0 (GraphPad Software LLC, La Jolla, CA). A Venn diagram was completed using Venn Diagram plotter software (https://omics.pnl.gov/software/venn-diagram-plotter) to illustrate the number of unique and shared genera across all samples evaluated. Response screening analysis was performed to identify genera with significantly higher and lower relative abundance in endodontic samples compared to sulcal samples using the fifty most abundant genera. For response screening analysis, False Discovery Rate (FDR) of <0.1 and *P*-value < 0.1 were used. The results were illustrated by graphs built using GraphPad Prism 8.2.0 using the mean relative abundance (MRA) and standard error of the mean (SEM). The relative abundance of OTUs in sulcal and endodontic samples were compared using two-way ANOVA (Analysis of Variance) in GraphPad Prism 8.2.0 (GraphPad Software LLC, La Jolla, CA). Bonferroni correction was used to adjust *P*-value; *P*-values < 0.05 were deemed statistically different. ANOVA was also used to compare samples regarding Chao1 and Shannon indices. In addition, principal component analysis (PCA) was performed based on significant genera identified by response screening analysis in order to compare samples and illustrate the genera associated with each category (i.e., endodontic vs. sulcal sample). Principal component analysis was performed using JMP Pro 11.

## Results

### Study Animals and Samples

A total of 10 endodontic and 10 sulcal samples were collected for analysis. The median age of sampled animals was 6 years [interquartile range (IQR) = 4.25; minimum = 2.5; maximum = 14]; the median body weight was 31.75 kg (IQR = 8.4 kg; minimum = 19.2 kg; maximum = 55.6 kg). The sex and reproductive status distribution of sampled dogs was 4 neutered males, 3 intact males, and 3 spayed females. The represented breeds were 1 Belgian Malinois, 2 German shepherd dogs, 1 German shepherd dog/Siberian Husky mix, 1 Siberian Husky, 2 mixed breed dogs, 1 Pit-bull mix dog, 1 Portuguese waterdog, and 1 Rottweiler. The sampled teeth included 4 maxillary canine teeth and 6 mandibular canine teeth. Radiographically evident apical periodontitis was present in all except 1 sampled tooth (E_D6). None of the animals had a clinically evident draining tract, soft tissue swelling, or cellulitis associated with the sampled tooth.

### Sequencing Results

All collected samples were amplified and sequenced using barcoded primers and next-generation sequencing of the V4 region of the 16 rRNA gene, median length per read of 301 bases was obtained. Quality filtered reads provided a total number of reads of 734,615; the average coverage was 36,730 reads per sample, standard deviation of 5,948; the number of reads per sample ranged from 23,903 to 49,303, with median of 35,357.

### Distribution and Comparison of Relative Abundance of Phyla and Genera

**Figure 1:**
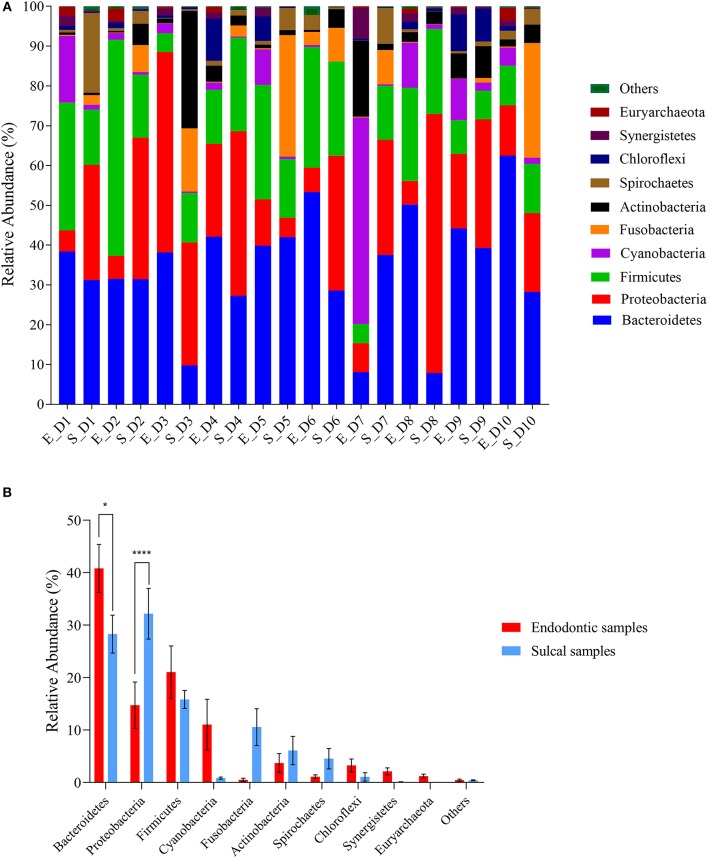
Relative abundance of phyla found in sulcal samples and endodontic samples from different dogs. **(A)** Relative abundance of the ten most abundant genera according to sulcal samples (S_) and endodontic samples (E_) from different dogs (D1 to D10); **(B)** Comparison between groups of samples using the most abundant phyla. Error bars represent standard error of the mean. The asterisks indicate significant difference between sulcal samples and endodontic samples (*Adjusted *P*-value 0.0125; ****Adjusted *P*-value < 0.0001).

The relative abundance of phyla is presented in Figure 1. The most abundant phyla in all samples were Bacteroidetes [sulcal samples (S_) = 28.29% ± 3.60%; endodontic samples (E_) = 40.80% ± 4.59%; *P*-value 0.0125], Proteobacteria (S_= 32.18% ± 4.82; E_= 14.73% ± 4.41%; *P*-value < 0.0001), and Firmicutes (S_= 15.83% ± 1.69%; E_= 21.03% ± 4.99%; *P*-value > 0.9999). The relative abundance of Bacteriodetes was significantly different between groups of samples, showing higher relative abundance in endodontic samples. On the other hand, significantly lower relative abundance of Proteobacteria was found in endodontic samples.

**Figure 2:**
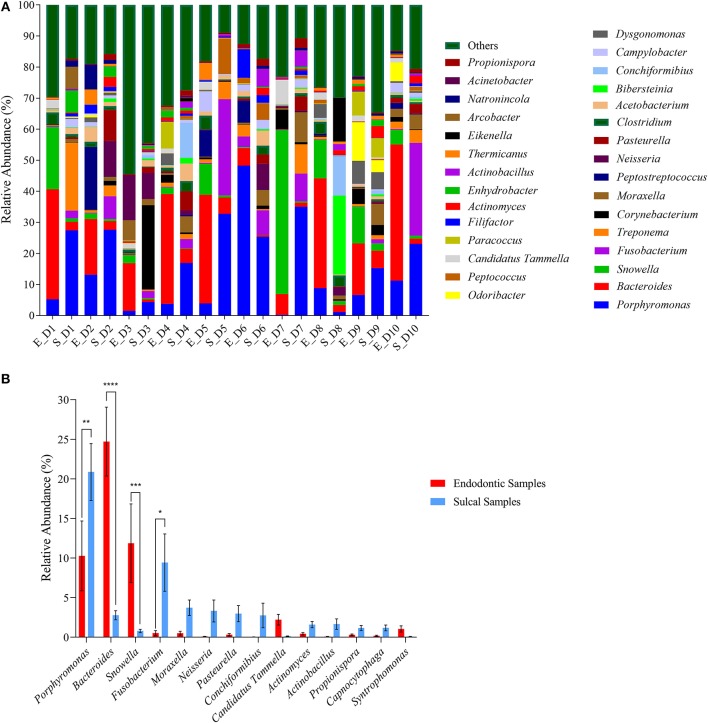
Relative abundance of genera found in sulcal samples and endodontic samples from different dogs. **(A)** Relative abundance of the thirty most abundant genera according to sulcal samples (S_) and endodontic samples (E_) from different dogs (D1 to D10); **(B)** Relative abundance of genera found as significant according to response screening analysis (*P*-value < 0.1; FDR *P*-value < 0.1; robust fit). Response screening analysis was performed using the fifty most abundant genera to identify significant taxa. Error bars represent standard error of the mean. The asterisks indicate significant difference between sulcal samples and endodontic samples (*Adjusted *P*-value 0.0119; **Adjusted *P*-value 0.0011; ***Adjusted *P*-value 0.0005; Adjusted *P*-value < 0.0001).

Regarding the bacterial genera identified, each sample presented a particular profile (Figure 2A). However, when response screening analysis and analysis of variance were performed, the relative abundance of the most abundant genera (*Porphyromonas, Bacteroides, Snowella*, and *Fusobacterium*) was statically different between endodontic and sulcal samples (Figure 2B). The relative abundance of *Bacteroides* was significantly higher in endodontic samples (S_= 2.77± 0.57%; E_= 24.70% ± 4.34%; *P*-value < 0.0001) and this genus was the most abundant in the respective samples. Additionally, *Snowella* (S_= 0.79% ± 0.21%; E_= 11.70% ± 4.96%; *P*-value = 0.0005) was significantly more abundant in endodontic samples compared to sulcal samples. On the other hand, *Porphyromonas* (S_= 20.86 ±11.36; E_= 10.27 ± 13.95; *P*-value 0.0011) and *Fusobacterium* (S_= 9.42 ± 11.46; E_= 0.51 ± 1.01; *P*-value 0.0119) were more abundant in sulcal samples when compared to endodontic samples.

**Figure 3:**
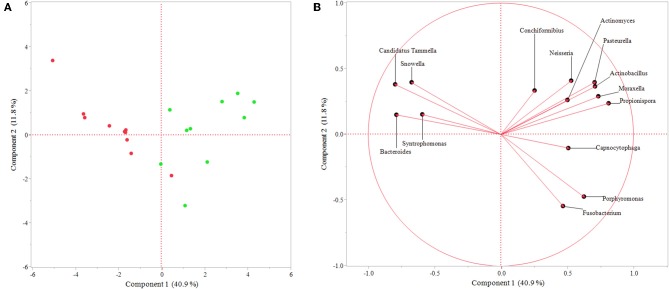
Principal component analysis of genera identified as significant by response screening analysis (*P*-value < 0.1; FDR *P*-value < 0.1; robust fit). **(A)** Graph showing similarity between samples represented by each point, which is colored regarding the site sampled (green = sulcal samples; red = endodontic samples); **(B)** Graph presenting genera correlated with endodontic samples (left side) and sulcal samples (right side). The variance explained by each component is shown in parentheses.

Additionally, PCA showed dissimilarity between sulcal and endodontic samples and the respective genera correlated with each category (Figure 3), corroborating the previous analysis. Importantly, the variance explained by component 1 is 40.9% and clearly separates genera according to the site sampled.

### Alpha Diversity and Venn Diagram

**Figure 4:**
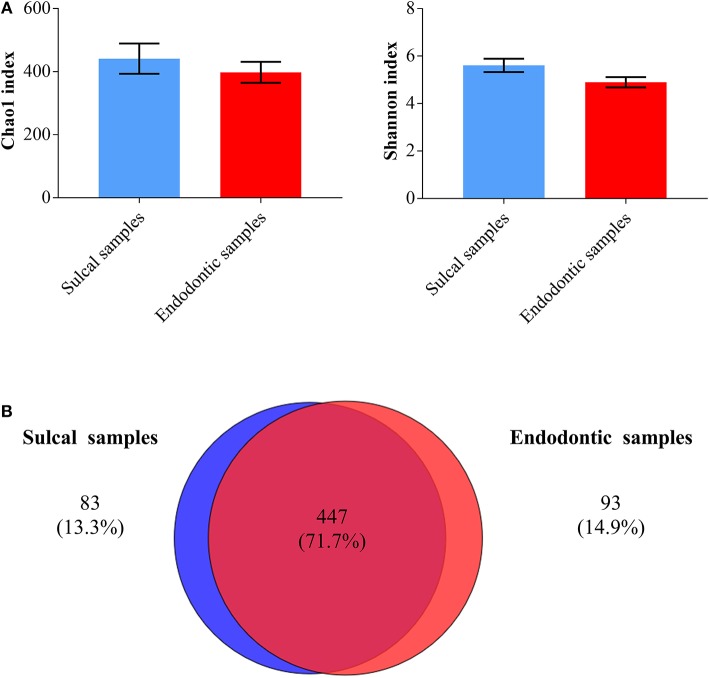
Bar graphs **(A)** illustrating Chao1 and Shannon indices found in sulcal samples and endodontic samples from dogs (significant difference not found, *P*-value ≤ 0.05). Venn diagram **(B)** illustrating the number of bacterial/archaeal genera shared (dark red overlap) and unique (blue circle = sulcal samples; red circle = endodontic samples) across all samples analyzed.

Chao1 and Shannon indices were calculated to assess richness (number of different OTUs in the samples) and diversity (how evenly the microorganisms are distributed in the samples), respectively. Endodontic samples and sulcal samples were similar regarding these indices (Figure 4; *P*-value ≤ 0.05). As shown by the Venn diagram, a greater proportion of genera were shared (71.7%) and the proportion of unique genera in each category of samples were notably close (sulcal samples = 13.3%; endodontic samples = 14.9%).

## Discussion

This is the first study to examine the microbiome of naturally occurring primary endodontic infections in dogs using a molecular approach, specifically 16S rRNA gene sequencing. We studied the endodontic contents of 10 non-vital periodontally healthy teeth with exposed pulps of client-owned dogs. All but one tooth had radiographic signs of apical periodontitis. Although a naturally-occurring disease model has some limitations (e.g., unknown duration of the pulp exposure, uncontrolled conditions), it provides the most realistic insight into the clinical scenario and therefore has the greatest potential for optimizing the diagnostic and treatment approaches. For comparison, subgingival plaque of the same teeth was sampled, subjected to the same analysis and the results compared to previously published studies (2, 5) to control our methodology approach.

Analysis of the samples revealed an abundant mixed microflora of comparable richness and diversity and with largely the same phyla obtained from both the sulcal and endodontic samples. The most abundant phyla in all samples were Bacteroidetes, Proteobacteria, and Firmicutes. These were previously reported to be the most common in subgingival plaque samples of healthy dogs (5) and endodontic samples obtained from non-vital teeth in dogs (8). The high similarity of taxa between the sulcal and endodontic samples may partially be attributed to the sampling method; despite extreme care while sampling the contents of the pulp cavity, it is possible that not isolating the tooth/coronal access with a dental dam may have resulted in contamination of the pulp cavity with sulcular bacteria. Interestingly, Rupf et al. (18) and Gomes et al. (19) have also reported similarities between the microbiota of periodontal and endodontic samples. However, their aims, methods, and patient selection were different to the ones described here. Namely, the teeth sampled in their studies were teeth with intact crowns affected by both periodontitis and endodontic disease, indicating a different portal for infection of the endodontic system (periodontal pocket vs. traumatic coronal pulp exposure). Moreover, further analysis of our data revealed significant differences between sulcal and endodontic samples in relative abundance in the most abundant phyla and genera. The relative abundance of Proteobacteria was significantly higher in sulcal samples. Specifically, *Porphyromonas, Fusobacterium, Moraxella, Neisseria, Pasteurella, Conchiformibius, Actimomyces, Actinobacillus Propionispora*, and *Capnocytophaga* were among the fifty most abundant genera in the sulcal samples, which is mostly in agreement with previously published studies on normal subgingival flora in periodontally healthy (i.e., no periodontitis) dogs (2, 5).

On the other hand, the relative abundance of Bacteriodetes was significantly higher in endodontic samples. Euryarchaeota was the unique Archaea phylum identified; future studies might help elucidate the possible role played by Archaea in the pathogenesis of endodontic disease in dogs. The *Bacteroides* genus was the most abundant in the respective samples, but *Snowella* was also significantly abundant in endodontic samples compared to sulcal samples. This is most likely a result of the endodontic environment favoring specific genera (11). Bacteriodetes/*Bacteroides* have been reported as one of the most frequently identified phyla/genera in the pulp cavities of teeth in dogs affected by primary endodontic infection (8, 10), but the other findings of the present study mostly differ from previously published data on endodontic microbiota in dogs. The observed difference may be a sequel of case selection. Namely, in one of the previous studies (identifying *Streptococcus, Propionibacterium, Staphylococcus, Neisseria*, and *Prevotella* as the most frequently isolated genera from the infected pulp cavities) was an experimental study with a defined duration of pulp exposure (9). Duration of pulp exposure most likely influences the endodontic microbiome. A decline in number of bacterial species with longer pulp exposure times was observed in one previous veterinary clinical study (10), while data from human literature show higher number and diversity of endodontic bacteria associated with the severity and acuteness of the endodontic disease (4, 18). Furthermore, the veterinary study (identifying *Pasteurella, Bacteroides*, and *Propionibacterium* as the most frequently isolated genera from the exposed pulp cavities) included both vital and non-vital fractured teeth (10). Finally, all previously published veterinary studies were performed using culture-based methods of bacterial identification (8–10), possibly missing some difficult-to-culture microorganisms (11, 20, 21).

In humans, Firmicutes and Bacteroidetes are the most commonly identified phyla associated with endodontic disease; Actinobacteria, Fusobacteria, Proteobacteria, Spirochaetes, Tenericutes, and Synergistetes are also commonly found (4, 6, 7). It is, however, difficult to directly compare the findings from dogs to those from humans as significant differences between canine and human oral microflora exist (3).

Although it is impossible to directly compare our results to those previously published with regards to Gram staining and oxygen requirements of bacteria involved in primary endodontic infections, it is still worth mentioning that the most abundant genera identified in our study are Gram negative strict anaerobes (data not found for *Snowella*), which would be expected in the necrotic endodontic environment. However, Ferreira et al. (9) reported predominance of Gram positive facultative anaerobic bacteria. Similarly, a high percentage of facultative anaerobic bacteria isolated from necrotic root canals was reported by Almansa Ruiz et al. (8), but not Srečnik et al. (10). Both previous clinical studies (8, 10) also reported on the ratio between Gram positive and Gram negative bacteria isolated from root canal samples to be almost 1:1, similar to the data from a study of non-vital teeth in cheetahs (20).

It is also worth mentioning that each sample in this study presented a particular genera profile. It has been shown in humans that composition of the endodontic microbiota differs consistently between individuals even with similar disease outcomes (4). On the contrary, certain microbes and features of the microbiota (4, 11) as well as endotoxin levels (22) in primary endodontic infections were related to differences in observed clinical and radiographic signs in humans. This was not confirmed in the previous study in dogs (10), while in this study only one tooth was lacking radiographic signs of apical periodontitis, making it impossible to draw any conclusions. However, the pulp cavity of this tooth (E_D6) had a different endodontic microbiome with high *Porphyromonas* relative abundance.

The passive sampling of the pulp cavity and sulcal contents could potentially yield mostly planktonic microorganisms, but as expected, this study confirmed that previous culture-based studies of primary endodontic infections in dogs (8–10) and cheetahs (20) underestimated the richness and diversity of the endodontic microbiota. Moreover, and contrary to previous reports, all endodontic samples in this study revealed bacteria. It is important to note that DNA sequencing techniques reveal all microorganisms present in the samples, viable, not viable, and viable but non-culturable, whereas, culture techniques reveal bacteria currently living in the endodontic system and likely contributing to infection. The 16S rRNA gene sequencing reveals the taxonomical composition of the microbiome, which may or may not explain pathogenesis, as it does not include the functional potential or expression profile (11). Therefore, further studies are warranted to identify putative endodontic pathogens, their relationship with other possible microorganisms, and the role of host response to better understand the pathogenesis of primary endodontic infections in dogs. Additionally, this cross-sectional study focused on the taxonomic composition exclusively in cases of non-vital teeth due to pulp exposure and additional studies could include how the taxonomic composition varies as disease progresses, or in cases of endodontic disease due to other causes (e.g., no pulp exposure or advanced caries) to optimize diagnostic and treatment strategies.

## Conclusion

Using a 16S rRNA gene sequencing approach to examine the microbiome of naturally occurring primary endodontic infections in dogs, we confirmed that the microbiome of the diseased endodontic system is comparably abundant with bacteria to the healthy subgingival plaque, with Bacteroidetes, Proteobacteria, and Firmicutes phyla prevailing in all samples. However, significant differences were found between sulcal and endodontic samples in relative abundance in the most abundant phyla and genera, which is most likely a result of the endodontic environment favoring establishment of certain bacteria.

## Data Availability Statement

The raw data supporting the conclusions of this manuscript will be made available by the authors, without undue reservation, to any qualified researcher.

## Ethics Statement

The animal study was reviewed and approved by Cornell University Institutional Animal Care and Use Committee (protocol number 2015-0117). Written informed consent was obtained from the owners for the participation of their animals in this study.

## Author Contributions

MR performed the DNA analysis and analyzed the results. MR, AN, NF, RB, and SP conceived the study. MR and RB conducted bioinformatics and data analysis. SP collected the samples. All authors reviewed the manuscript.

### Conflict of Interest

The authors declare that the research was conducted in the absence of any commercial or financial relationships that could be construed as a potential conflict of interest.
